# Evaluation of Vaccine Safety After the First Public Sector Introduction of Typhoid Conjugate Vaccine—Navi Mumbai, India, 2018

**DOI:** 10.1093/cid/ciab059

**Published:** 2021-01-27

**Authors:** Ashley T Longley, Kashmira Date, Stephen P Luby, Pankaj Bhatnagar, Adwoa D Bentsi-Enchill, Vineet Goyal, Rahul Shimpi, Arun Katkar, Vijay Yewale, Niniya Jayaprasad, Lily Horng, Abhishek Kunwar, Pauline Harvey, Pradeep Haldar, Shanta Dutta, Jane F Gidudu

**Affiliations:** 1 National Foundation for the Centers for Disease Control and Prevention, Atlanta, Georgia, USA; 2 Global Immunization Division, Center for Global Health, Centers for Disease Control and Prevention, Atlanta, Georgia, USA; 3 Infectious Diseases and Geographic Medicine, Stanford University, Stanford, California,USA; 4 World Health Organization–Country Office for India, National Public Health Surveillance Project, New Delhi, India; 5 Department of Immunization, Vaccines and Biologicals, World Health Organization, Geneva, Switzerland; 6 Dr Yewale Multi Specialty Hospital for Children, Navi Mumbai, India; 7 Ministry of Health and Family Welfare, Government of India, New Delhi, India; 8 National Institute of Cholera and Enteric Diseases, Indian Council of Medical Research, Kolkata, India

**Keywords:** adverse events following immunization, typhoid vaccination campaign, typhoid conjugate vaccine, adverse events of special interest

## Abstract

**Background:**

In December 2017, the World Health Organization (WHO) prequalified the first typhoid conjugate vaccine (TCV; Typbar-TCV). While no safety concerns were identified in pre- and postlicensure studies, WHO’s Global Advisory Committee on Vaccine Safety recommended robust safety evaluation with large-scale TCV introductions. During July–August 2018, the Navi Mumbai Municipal Corporation (NMMC) launched the world’s first public sector TCV introduction. Per administrative reports, 113 420 children 9 months–14 years old received TCV.

**Methods:**

We evaluated adverse events following immunization (AEFIs) using passive and active surveillance via (1) reports from the passive NMMC AEFI surveillance system, (2) telephone interviews with 5% of caregivers of vaccine recipients 48 hours and 7 days postvaccination, and (3) chart abstraction for adverse events of special interest (AESIs) among patients admitted to 5 hospitals using the Brighton Collaboration criteria followed by ascertainment of vaccination status.

**Results:**

We identified 222/113 420 (0.2%) vaccine recipients with AEFIs through the NMMC AEFI surveillance system: 211 (0.19%) experienced minor AEFIs, 2 (0.002%) severe, and 9 serious (0.008%). At 48 hours postvaccination, 1852/5605 (33%) caregivers reported ≥1 AEFI, including injection site pain (n = 1452, 26%), swelling (n = 419, 7.5%), and fever (n = 416, 7.4%). Of the 4728 interviews completed at 7 days postvaccination, the most reported AEFIs included fever (n = 200, 4%), pain (n = 52, 1%), and headache (n = 42, 1%). Among 525 hospitalized children diagnosed with an AESI, 60 were vaccinated; no AESIs were causally associated with TCV.

**Conclusions:**

No unexpected safety signals were identified with TCV introduction. This provides further reassurance for the large-scale use of Typbar-TCV among children 9 months–14 years old.

## Teaser

Using data from passive surveillance, telephone interviews, and active surveillance for adverse events of special interest, no safety signals were identified from the first public sector introduction of Typbar-TCV among children 9 months–14 years old in Navi Mumbai, India.

Typhoid fever, a vaccine-preventable disease, results in nearly 11 million illnesses and 116 800 deaths annually [1]. The World Health Organization (WHO) recommends the use of typhoid vaccines in addition to water and sanitation improvements for typhoid prevention and control [2]. Previously licensed safe and effective vaccines are available, but these cannot be administered to children younger than 2 years old. Newer typhoid conjugate vaccines (TCVs) are now available for children as young as 6 months old [2]. Typbar-TCV (Bharat Biotech International Limited, India), in which the *Salmonella* Typhi Vi capsular polysaccharide antigen is conjugated to the tetanus toxoid carrier protein (Vi-TT), was licensed in India in 2013 as a single-dose intramuscular vaccine for use in persons aged 6 months–45 years old.

In December 2016, WHO’s Global Advisory Committee on Vaccine Safety reviewed available safety data on Typbar-TCV. In the pre- and postlicensure studies and early postmarketing surveillance, no safety concerns were identified, although the sample sizes were small and the surveillance data were largely obtained passively. To ensure the continued safety of vaccines and because vaccine safety concerns have the potential to impede effective immunization activities, surveillance for adverse events following immunization (AEFIs) is critical to sustaining confidence in immunization programs [3]. The committee concluded that more robust safety data were required, recommending TCV introductions include safety evaluations using larger sample sizes and methods for data standardization [4]. With larger cohorts, adverse events of special interest (AESIs) should be predefined, monitored, and, if detected, assessed for causal association with the vaccine [5]. The Brighton Collaboration criteria are globally accepted AEFI case definitions enabling standardization and comparability of safety data in such evaluations [4, 6, 7].

In October 2017, the WHO Strategic Advisory Group of Experts on Immunization recommended the use of TCVs for typhoid control, followed by WHO’s prequalification of Typbar-TCV in December 2017 [8]. In 2018, the Navi Mumbai Municipal Corporation (NMMC), the local government body in the city of Navi Mumbai, India, was the first in the world to introduce TCV into a public sector immunization program. The vaccine was introduced in a phased campaign for children aged 9 months–14 years old, the first phase of which was conducted from July to August 2018. The second phase was planned for 2020, to be followed by TCV introduction into the routine immunization program. During the first phase, 113 420 children received TCV according to administrative reports [9], providing an opportunity to conduct a comprehensive safety evaluation. We describe the safety profile of Typbar-TCV using passive and active surveillance approaches to report AEFIs and investigate AESIs using the Brighton Collaboration definitions.

## METHODS

### Definitions

According to the WHO guidelines, an AEFI is any untoward medical occurrence following immunization that does not necessarily have a causal relationship with vaccine usage. Serious AEFIs result in death or persistent or significant disability, are life-threatening, or require hospitalization or prolonging of existing hospitalization [10]. Clusters of cases or cases resulting in community/media concern are also deemed serious, as these can negatively affect the immunization program. Nonserious AEFIs, or minor AEFIs, pose little threat to the vaccine recipient, usually occur within a few hours to days of vaccination, and resolve shortly thereafter. Severe reactions, describing the clinical severity of an event, can be disabling but may not be life-threatening and do not result in long-term complications [4, 11, 12].

### Routine AEFI Surveillance

The NMMC follows the Government of India’s national AEFI surveillance guidelines. Each vaccination clinic maintains a logbook of AEFIs reported within 30 days following any vaccination. All clinics report AEFIs monthly to the NMMC, including the submission of nil reports, when applicable. The reporting forms are then submitted monthly to the national level. Severe or serious AEFIs are reported immediately from the facility to the district level; the district reports simultaneously to the state and the national Immunization Division for investigation [12].

Vaccination clinic staff passively reported AEFIs within 30 days of TCV vaccination to the Reproductive Child Health Officer of NMMC using the existing AEFI surveillance system. Local health officials classified AEFIs as minor, severe, or serious, per the Indian guidelines, adapted from the WHO guidelines [10]. We tallied the reported AEFI information, including classification, date of AEFI onset, age of vaccine recipient, date and location of vaccination, and hospitalization.

### Active Surveillance by Telephone Interviews for Nonserious AEFIs

We conducted active surveillance for nonserious AEFIs among a sample of vaccine recipients at 48 hours and again at 7 days following TCV vaccination. Vaccination cards for the TCV campaign were printed in duplicate, one for the vaccine recipient and another kept at the vaccination site. On the cards kept at the vaccination site, vaccinators recorded the date of vaccination; the child’s name, age, and address; and up to 2 telephone numbers of caregivers. Interviewers reviewed all vaccination cards daily from each vaccination site. Of the cards with a telephone number, interviewers systematically sampled every 14th card to obtain a sample of 7% of vaccine recipients from each site daily to obtain a geographic and temporally representative sample (5% to generate a reasonable sample size and an additional 2% to account for nonresponse). Using a standard questionnaire, caregivers were interviewed by telephone in local languages at the 2 time points following vaccination. Nonserious AEFIs solicited at each telephone call included a list of prespecified conditions: fever, injection site pain, swelling and/or redness, induration, pustule with discharge, malaise, headache, vomiting, nausea, diarrhea, persistent crying, and myalgia. Interviewers also documented any other nonspecified conditions reported.

### Active Surveillance for AESIs

Last, we conducted hospital-based active surveillance at 5 hospitals in Navi Mumbai to identify AESIs, including anaphylaxis, Guillain-Barré syndrome, aseptic meningitis, encephalitis, myelitis, acute disseminated encephalomyelitis, seizures, thrombocytopenia (<150 000 platelets/μL of blood), and sudden unexplained death. These conditions were identified as a subset of rare but serious AEFIs known to occur following administration with other conjugate vaccines and for which Brighton Collaboration case definitions have been validated [13–18].

The selected hospitals were sites with existing typhoid disease surveillance as part of an overall TCV evaluation (Table 1). Surveillance for AESIs began 1 week before the vaccination campaign and continued for 42 days after the last day of the campaign. One study physician at each hospital reviewed medical charts daily of all children aged 9 months–14 years old identified in emergency departments or admitted for any duration in inpatient wards or intensive care units, regardless of vaccination status. We included AESI cases within the vaccine-eligible age group in the absence of vaccination to understand the background occurrence of these conditions in the general population.

**Table 1.** **Characteristics of the Hospitals Conducting Surveillance for Adverse Events of Special Interest for the Typhoid Conjugate Vaccine Safety Evaluation—Navi Mumbai, India, 2018**

Hospital Name	Bed Capacity	Facility Type
Mahatma Gandhi Mission (MGM), New Bombay Hospital, Vashi	180 beds	Private hospital
D.Y. Patil Medical College and Hospital	1500 beds	Private medical college (part charitable, part paid)
Navi Mumbai Municipal Corporation (NMMC) General Hospital—Vashi; First Referral Unit (FRU)	400 beds	Public hospital
Mathadi Trust Hospital	120 beds	Trust hospital for Mathadi workers and their families
Dr Yewale Multispecialty Hospital for Children	40 beds	Private pediatric hospital

If AESIs were documented in the charts at any time during hospitalization, study physicians used condition-specific chart-abstraction tools to extract relevant information from the medical charts to determine if the event met the Brighton Collaboration case definition. The study physicians then further characterized the case definitions into levels of diagnostic certainty—from level 1, the highest level of specificity, to level 3, the lowest level of specificity for the respective event [6]—then ascertained vaccination status from vaccination cards, the vaccine registry, or from patient/caregiver verbal reports obtained via the treating physicians. Chart-abstraction tools were finalized upon patient discharge and data were entered into a central electronic database.

### Data Analysis

We described the frequencies of reported and identified adverse events during the periods of surveillance for each surveillance method. Rates of reported AEFIs were calculated per TCV dose administered and classified according to the Government of India’s guidelines for minor, severe, and serious events [12]. A *P* value of less than .05 was considered significant when performing chi-square tests.

### Ethical Approval

This evaluation protocol was approved by the Centers for Disease Control and Prevention and the institutional ethics committees of the Indian Council of Medical Research–National Institute of Cholera and Enteric Diseases, WHO, Stanford University, and site hospitals (D.Y. Patil, MGM New Bombay Hospital, and an independent ethics committee) as an assessment of a public health program.

## RESULTS

**Figure 1.**
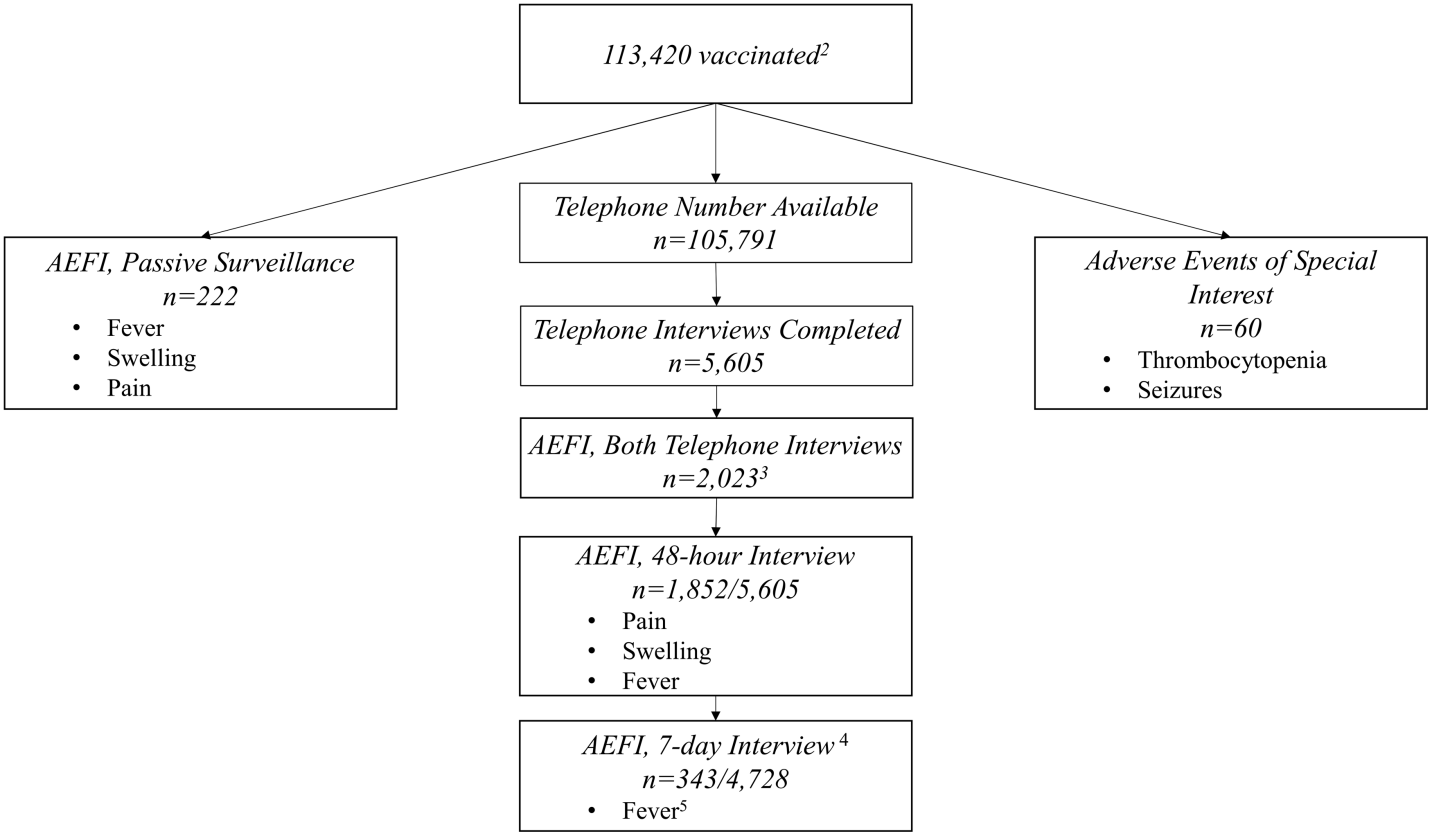
Most common safety-related events by assessment method among individuals who were reported to have received TCV^1^—Navi Mumbai, India, 2018. ^1^Typbar-TCV, Bharat Biotech International Limited, India. ^2^Per administrative reports. ^3^A total of 2023 children experienced at least 1 AEFI at either time point (48 hours or 7 days following TCV vaccination). At 7 days following vaccination, 172 of 343 children had reported an AEFI at 48 hours as well. ^4^Telephone interviews were conducted among 5605 caregivers of TCV recipients at 48 hours after vaccination. At 7 days following TCV vaccination, 4728 of those caregivers were interviewed again. ^5^Fever was the only AEFI reported among more than 1% of vaccine recipients 7 days following vaccination. Abbreviations: AEFI, adverse events following immunization; TCV, typhoid conjugate vaccine.

Of the 113 420 children who reportedly received TCV, 222 experienced an AEFI as identified via the passive routine surveillance system, 2023 experienced a nonserious AEFI as reported during telephone interviews, and 60 experienced an AESI as identified through the hospital-based surveillance (Figure 1).

### Routine AEFI Surveillance

A total of 222/113 420 (0.2%) vaccine recipients were identified from the government's passive surveillance system with at least 1 AEFI following TCV vaccination: 211 (0.19%) children experienced minor AEFI, 2 (0.002%) severe AEFI, and 9 (0.008%) experienced serious AEFI. An injection abscess and the inability to walk were the only reported severe events, although neither condition required hospitalization. No follow-up information was available for these 2 children. Nine children were hospitalized, and these cases were deemed serious AEFIs per the national guidelines. Reasons for hospitalization documented in the logbooks included fever, swelling, rash, itching, and/or giddiness. The median age of children experiencing an AEFI was 6 years (range, 10 months–15 years), including 6 children, all 15 years old, vaccinated outside of the target age group. The overall reporting rate of AEFIs was 195.7 per 100 000 children vaccinated (Table 2). Fever (n = 143) and swelling (n = 37) were the most reported events, with a median onset 2 days following vaccination (range, 0–24 and 0–20 days, respectively) (Supplementary Figure 1). Most fevers occurred among children aged 2–10 years old (Table 3).

**Table 2.** **Rates of Adverse Events Following Immunization (AEFIs) Reported Within 30 Days of Vaccination With Typhoid Conjugate Vaccine Via the Passive AEFI Surveillance System—Navi Mumbai, India, 2018**

AEFIs	n	Rate^a^
Total number of children reporting 1 or more AEFI	222	195.7
Minor^b^	211	186.0
Fever	143	126.1
Swelling	37	32.6
Pain	23	20.3
Rash	15	13.2
Vomiting	12	10.6
Giddiness	6	5.3
Body aches	3	2.6
Itching	3	2.6
Headache	2	1.8
Cough/cold	2	1.8
Severe	2	1.8
Abscess	1	0.9
Inability to walk	1	0.9
Serious	9	7.9
Hospitalized^c^	9	7.9

^a^Per 100 000 vaccinated.

^b^246 minor AEFI were reported among 211 children.

^c^Reasons for hospitalization included fever, swelling, rash, itching, and/or giddiness.

**Table 3.** **Age Distribution of Children With Reported Fevers After Vaccination With Typhoid Conjugate Vaccine Identified Through Passive Surveillance and by the Telephone Interviews—Navi Mumbai, India, 2018**

		Telephone Interviews, n (%)	
Age Group	Passive Surveillance (n = 141^a^), n (%)	48 Hours (n = 416)	7 Days (n = 200)
<2 Years	21 (15)	56 (14)	26 (13)
2–5 Years	49 (35)	121 (29)	64 (32)
6–10 Years	44 (31)	134 (32)	58 (29)
11–15 Years	27 (19)	105 (25)	52 (26)

^a^Age unknown for 2 children with reported fever.

### Active Surveillance by Telephone Interviews for Nonserious AEFIs

Overall, 93% (105 791/113 420) of caregivers of vaccine recipients had a telephone number available on the TCV vaccination cards. Interviewers contacted 5.8% (6139/105 791) of these caregivers at 48 hours following vaccination. Overall, 91.3% (5605/6139) of caregivers contacted participated in the first interview, for a total of 4.9% (5605/113 420) of caregivers of all vaccine recipients. Of those interviewed, 53% (2976/5605) of children were male, and the median age was 8 years (range, 3 months–15 years). Eighty-eight children were outside of the target age group; 4 were younger than 9 months old and 84 were 15 years old. A total of 33% (1852/5605) of children experienced at least 1 AEFI within 48 hours following vaccination; 51% (n = 945) were male and the median age was 8 years (range, 3 months–15 years). The most reported AEFIs at this time included pain (1452/5605, 26%), swelling (419/5605, 7.5%), and fever (416/5605, 7.4%) (Table 4).

**Table 4.** **Adverse Events Following Immunization Reported by Caregivers at 48 Hours and 7 Days Following Vaccination With Typhoid Conjugate Vaccine, by Condition—Navi Mumbai, India, 2018**

	48 Hours (n = 5605)	7 Days (n = 4728)
Total number (%) of children reporting 1 or more AEFI	1852 (33)	343 (7)
Conditions, n (%)		
Local reactions		
Pain	1452 (26)	52 (1.1)
Swelling	419 (7.5)	21 (0.4)
Redness	117 (2.1)	11 (0.2)
Induration	56 (1.0)	4 (0.1)
Pustule with discharge	18 (0.3)	7 (0.1)
Systemic reactions		
Fever	416 (7.4)	200 (4.2)
Malaise	14 (0.2)	5 (0.1)
Headache	61 (1.1)	42 (1)
Persistent crying	26 (0.5)	5 (0.1)
Myalgia	26 (0.5)	14 (0.3)
Gastrointestinal reactions		
Vomiting	54 (1.0)	38 (0.8)
Nausea	14 (0.2)	12 (0.3)
Diarrhea	43 (0.8)	26 (0.5)
Othera	20 (0.4)	35 (0.7)
Total AEFIs	2736	508

Abbreviation: AEFI, adverse event following immunization.

^a^Other conditions were reported in <10 children, included itching, cold-like symptoms, body aches, chills, etc.

We attempted to contact the caregivers of all 5605 vaccine recipients again 7 days following vaccination, and successfully interviewed 4728 (84%). At the second interview, 7.3% (343/4728) of children had experienced or continued to experience 1 or more AEFI. Among these children, 171 (50%) had reported no AEFI at the 48-hour interview. The most reported AEFI on day 7 was fever (200/4728, 4.2%); all other events were reported by 1% or fewer of the vaccine recipients (Table 4).

At both time points, fever was most common among children older than 2 years old, with similar proportions among the older age groups (Table 3).

### Active Surveillance for AESIs

A total of 555 AESIs were identified among 525 children, 60 (11%) of whom presented within 42 days of receiving TCV. Thrombocytopenia (n = 365) and seizures (n = 160) were the most common events (Table 5). No statistically significant differences were observed for thrombocytopenia (*P* = .73) or seizures (*P* = .96) between children who had received TCV and those who had not.

**Table 5.** **Adverse Events of Special Interest Identified During Hospital-Based Surveillance, by Typhoid Conjugate Vaccine Vaccination Status—Navi Mumbai, India, 2018**

	Unvaccinated	Vaccinated
Total number of children reporting 1 or more AESI (n = 525)	465	60
Event of special interest,^a^ n (%)		
Thrombocytopenia	322 (69)	43 (72)
Seizure	142 (31)	18 (30)
Encephalitis	8 (2)	0 (0)
Acute disseminated encephalomyelitis	7 (2)	0 (0)
Meningitis	3 (1)	0 (0)
Anaphylaxis	3 (1)	0 (0)
Myelitis	2 (<.1)	0 (0)
Guillain-Barré syndrome	1 (<.1)	1 (2)
Sudden unexplained death	1 (<.1)	0 (0)
Hypersensitivity, non-anaphylaxis	4 (1)	0 (0)
Total number of AESIs (n = 555)	493	62

Abbreviation: AESI, adverse event of special interest.

^a^Percentage of children experiencing each AESI; a child could experience 1 or more AESI; therefore, total percentages do not equal 100%.

Among vaccinated children with an AESI, 43 had thrombocytopenia, 18 had seizures, and 1 child was suspected to have Guillain-Barré syndrome. The interval between vaccination and thrombocytopenia onset was 5–39 days (median, 23 days) and between vaccination and seizure onset was 0–36 days (median, 18 days). Thirty-seven cases met the Brighton Collaboration diagnostic certainty level 1 for thrombocytopenia and 6 were classified as level 2. Of the seizure cases, one met the level 1 criteria, one was categorized as level 2, and one was categorized as level 3. Two cases did not meet the Brighton Collaboration case definition due to insufficient information, and the remaining 13 physician-diagnosed seizures did not meet the criteria upon further review of the medical charts. Similarly, the case of suspected Guillain-Barré syndrome did not meet the Brighton Collaboration case definition upon further investigation (Supplementary Table 1).

Over half of the vaccinated children with thrombocytopenia had a final diagnosis of dengue fever (23/43, 54%); 83% (19/23) of these diagnoses were laboratory confirmed per the medical charts. Other common diagnoses included acute febrile illness (8/43, 19%), other viral fevers (5/43, 12%), and malaria (4/43, 9.3%). The final diagnoses among the unvaccinated children with thrombocytopenia were similar, most commonly dengue fever (131/322, 41%), acute febrile illness (52/322, 16%), other viral fevers (37/322, 12%), and malaria (30/322, 9.3%).

The final diagnoses among the 3 seizure cases meeting the Brighton Collaboration case definitions were epilepsy (n = 2) and malaria (n = 1). Most incidents of seizure among vaccinated children were diagnosed as febrile seizures (13/18, 72%) and were among children aged 5 years old or younger, as were the seizures among unvaccinated children (96/142, 68%).

No deaths were recorded among those who received TCV. The case presenting with Guillain-Barré syndrome was investigated by the NMMC AEFI Committee; however, no other AESI required additional investigations.

### Case Summary—Guillain-Barré Syndrome

A 2-year-old female experienced decreased tone in both lower limbs on the day of TCV vaccination, according to caregiver reports. The vaccination clinic staff referred her to the hospital, where investigations were conducted, including a nerve conduction velocity test and electromyography. The provisional diagnosis of Guillain-Barré syndrome was subsequently ruled out, but she remained under investigation for acute flaccid paralysis following vaccination. She was referred to a tertiary hospital in Mumbai where several investigations were advised but not conducted due to additional caregiver costs. The AEFI Committee was unable to complete the causality assessment due to insufficient information. The child fully recovered and was discharged within 2 weeks.

## DISCUSSION

Our findings from the postintroduction evaluation of TCV vaccine safety in Navi Mumbai contribute important data on the safety of Typbar-TCV. We used a combination of passive and active surveillance approaches to evaluate nonserious and serious AEFIs. The safety data described here support findings from the TCV clinical trials and early postmarketing surveillance reviewed by the Global Advisory Committee on Vaccine Safety, indicating that Typbar-TCV is a safe vaccine when administered to children aged 9 months–14 years old [4, 19].

The most frequently reported AEFIs to the passive surveillance system and through telephone interviews were mild and resolved within 1 week. The most common presentations—fever, swelling, and injection site pain—are consistent with AEFIs associated with the Vi polysaccharide typhoid vaccine [20, 21], and also similar to data from TCV clinical trials [22–24] and from an emergency vaccination campaign with Typbar-TCV in Pakistan [25]. Local reactions were most commonly reported by caregivers, as is expected with parenteral vaccines [26–29]. Additionally, vaccine conjugation with tetanus toxoid, as in Typbar-TCV, has been shown to result in higher rates of local reactions than other conjugated vaccines [26, 30]. Similarly, fever is one of the most commonly reported AEFI among routine immunizations [31], and we found that no age group was disproportionately affected. While the reported fevers were temporally associated with vaccination, it is important to consider common etiologies of fever in the Navi Mumbai setting, especially during the monsoon season (eg, dengue fever and malaria), when interpreting the results from caregiver reports. In addition to minor AEFIs, 9 children were hospitalized because of symptoms deemed serious by the NMMC health officials. While hospital admission was advised out of an abundance of caution (NMMC, personal communication, 2018), these events were classified as serious under the WHO and Indian AEFI surveillance guidelines [10, 12].

Thrombocytopenia was observed among both TCV vaccine recipients and nonrecipients who were hospitalized. The final diagnoses were similar between groups, suggesting that the high proportion of thrombocytopenia among all patients identified at these hospitals was a result of infectious diseases (eg, dengue fever) and not due to vaccination with TCV. Most seizure cases identified from the hospital-based surveillance were febrile seizures occurring among children aged 5 years or younger, which is consistent with the literature on febrile seizures after the administration of other vaccines in younger children [32, 33]. The occurrence of AESIs among the unvaccinated population is an indication that there is an expected frequency of these events within the vaccine-eligible age group, and this should be considered when assessing causality. For example, the period of surveillance coincided with the peak of the dengue fever epidemic season, during which time higher rates of thrombocytopenia were expected due to the well-described association between thrombocytopenia and dengue infection [34].

This evaluation was subject to limitations. While no other vaccines were administered simultaneously during the campaign, if children in this cohort received other vaccinations within 30 days of TCV vaccination, we did not capture that information. A general limitation of passive surveillance is the risk of underreporting. However, because this was a new vaccine, there was the potential for overreporting of serious events, such as advising hospitalization as a precautionary measure. The Government of India’s guidelines report events as minor, severe, and serious, whereas other systems may only categorize events as nonserious and serious. While our findings were not affected, this may limit comparability with future AEFI studies. As we solicited AEFIs by telephone, there was the potential for overreporting, and all events were reported by caregivers without objective verification of temperature or event by healthcare workers. We believe that the impact of these limitations is low because the fever rates identified are comparable to those reported from administration with other vaccines. All final diagnoses and laboratory results for AESI cases were obtained from medical charts. A future evaluation with laboratory testing would be beneficial to corroborate our dengue infection findings in children with thrombocytopenia. Last, the occurrence of AESIs cannot be generalized to the vaccinated population because AESIs were captured only among those who sought care at the study site hospitals, and the proportion of vaccine recipients who sought care at these hospitals is unknown. However, by documenting AESIs among unvaccinated children, we were able to observe that the prespecified conditions did not occur more frequently among vaccinated children than among those who were unvaccinated and were therefore likely coincidental events expected in the general population.

### Conclusions

Using a multipronged approach to conduct a safety evaluation including passive and active surveillance, we did not identify any unexpected safety signals among this large cohort of TCV recipients. This provides further reassurance that Typbar-TCV is safe for children aged 9 months–14 years old. While future evaluations should assess the safety of TCV in special populations, including malnourished children and pregnant women, our results suggest that there is no safety concern that would limit vaccine deployment. Additionally, the use of surveillance tools such as the Brighton Collaboration criteria may be of value to subsequent TCV and other new vaccine introductions with large target populations.

## Supplementary Data

Supplementary materials are available at *Clinical Infectious Diseases* online. Consisting of data provided by the authors to benefit the reader, the posted materials are not copyedited and are the sole responsibility of the authors, so questions or comments should be addressed to the corresponding author.

ciab059_suppl_Supplementary_Figure_S1

ciab059_suppl_Supplementary_Material

## Notes

***Acknowledgments.*** We thank the following organizations and individuals for their contributions to this evaluation: the Navi Mumbai Municipal Corporation leadership and staff, the Government of India Ministry of Health and Family Welfare Expanded Program on Immunization, State of Maharashtra Department of Public Health and Family Welfare, Indian Academy of Pediatrics Navi Mumbai Chapter, Bharat Biotech International Limited, the Indian Council of Medical Research, WHO headquarters (Dr Patrick Zuber), WHO–South East Asia Regional Office (Dr Sunil Bahl), WHO-India National Public Health Surveillance Project, the Navi Mumbai Municipal Corporation Hospital (Dr Savita Daruwalla, Dr Yashank Yewale), D.Y. Patil Medical College and Hospital (Dr Rajesh Rai, Dr Amit Saxena), Mathadi Trust Hospital (Dr Divya Warrier), MGM New Bombay Hospital Vashi (Dr Jeetendra Gavhane, Dr Alpa Bhosale), and Dr Yewale Multi Specialty Hospital for Children (Dr Dhanya Dharmapalan, Dr Asmita Patil).

***Financial support.*** This work was co-funded by the Bill & Melinda Gates Foundation (grant number OPP1169264) to Stanford University.

***Potential conflicts of interest.*** The authors: No reported conflicts of interest. All authors have submitted the ICMJE Form for Disclosure of Potential Conflicts of Interest. Conflicts that the editors consider relevant to the content of the manuscript have been disclosed.
