# Supplementary figures and images for: Evaluation of Vaccine Safety After the First Public Sector Introduction of Typhoid Conjugate Vaccine—Navi Mumbai, India, 2018
Source: Clin Infect Dis. 2021 Jan 27;73(4):e927–33. doi: 10.1093/cid/ciab059 (PMC8366822; doi:10.1093/cid/ciab059)

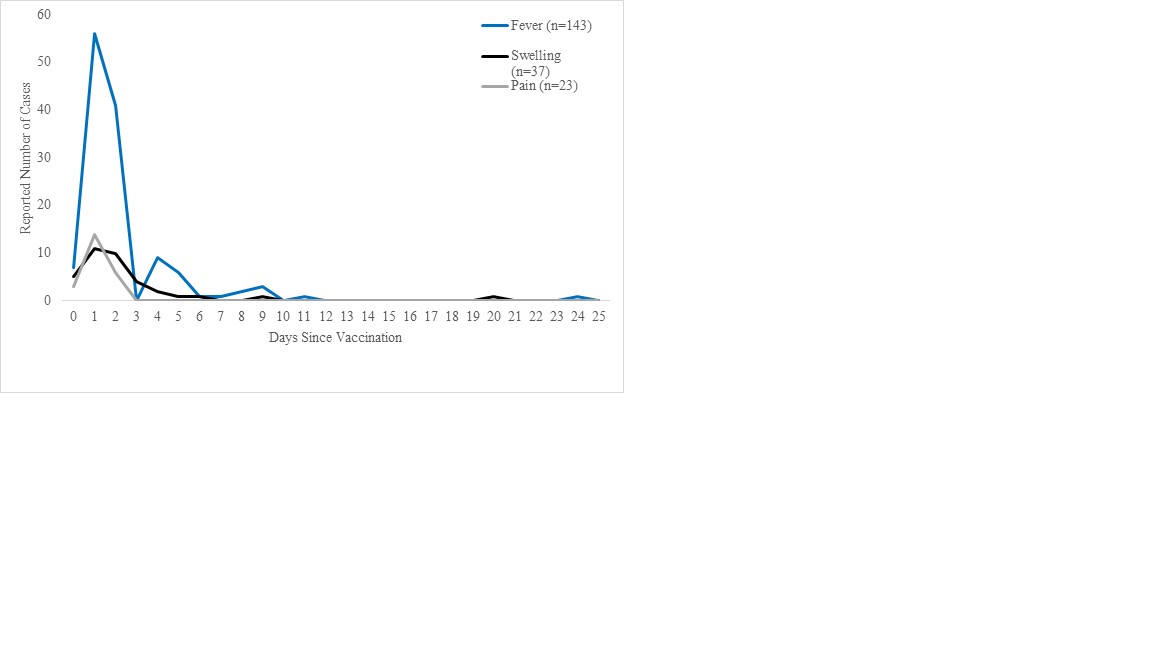

Supplement: ciab059_suppl_Supplementary_Figure_S1 [file ciab059_suppl_supplementary_figure_s1.jpeg]
