# Supplementary material for: Evaluation of Vaccine Safety After the First Public Sector Introduction of Typhoid Conjugate Vaccine—Navi Mumbai, India, 2018
Source: Clin Infect Dis. 2021 Jan 27;73(4):e927–33. doi: 10.1093/cid/ciab059 (PMC8366822; doi:10.1093/cid/ciab059)
Supplement: ciab059_suppl_Supplementary_Material [file ciab059_suppl_supplementary_material.docx]

**Supplementary Table 1. Brighton Collaboration level of diagnostic certainty^1^ of adverse events of special interest (AESI) among children vaccinated with typhoid conjugate vaccine (n=60)^2^—Navi Mumbai, India, 2018**

| Event | Level 1 | Level 2 | Level 3 | Category 4 | Category 5 |
| --- | --- | --- | --- | --- | --- |
| Thrombocytopenia | 37 | 6 | N/A | 0 | 0 |
| Seizure | 1 | 1 | 1 | 2 | 13 |
| Guillain-Barré syndrome | 0 | 0 | 0 | 0 | 1 |

^1^Level 1: Meets all the diagnostic certainty criteria; highest level of diagnostic specificity, least sensitive

Level 2: Intermediate level of specificity, lower sensitivity for respective AEFI

Level 3: Lower level of specificity, highly sensitive for respective AEFI

Category 4: Insufficient evidence

Category 5: Not a case

^2^A child could experience 1 or more AESI

**Supplementary Figure Legend**

**Supplementary Figure 1. Number of children with reported fever, swelling, or injection site pain by days since vaccination with typhoid conjugate vaccine via the passive adverse events following immunization (AEFI) surveillance system—Navi Mumbai, India, 2018**
